# Regional Trends and Socioeconomic Predictors of Adolescent Pregnancy in Nigeria: A Nationwide Study

**DOI:** 10.3390/ijerph19138222

**Published:** 2022-07-05

**Authors:** Blessing Jaka Akombi-Inyang, Emma Woolley, Chinonyerem Ogadi Iheanacho, Khulan Bayaraa, Pramesh Raj Ghimire

**Affiliations:** 1 School of Population Health, University of New South Wales, Sydney, NSW 2052, Australia; 2 School of Education, Macquarie University, Macquarie Park, NSW 2109, Australia; emma.woolley@ymail.com; 3 Department of Clinical Pharmacy and Public Health, University of Calabar, Calabar 540271, Nigeria; coiheanacho@unical.edu.ng; 4 Department of Obstetrics and Gynaecology, Intermed Hospital, Ulaanbaatar 17040, Mongolia; khulanbayaraa11@gmail.com; 5 Ujyalo Nepal, Ratnanagar 44200, Nepal; prameshraj@hotmail.com

**Keywords:** adolescent pregnancy, teenage pregnancy, Nigeria, sexual and reproductive health, adolescent health, trend

## Abstract

Adolescent pregnancy is a major health concern which has lifelong consequences. The aim of this study is to examine the regional trends and socioeconomic predictors of adolescent pregnancy in Nigeria. This study used pooled data from the 2008, 2013 and 2018 Nigeria Demographic and Health Survey (NDHS). A total of 22,761 women aged 15–19 years were selected across the three surveys. Multilevel logistic regression analysis that adjusted for cluster and survey weights was used to identify predictors of adolescent pregnancy in Nigeria, across the six geopolitical zones of Nigeria. Adolescent pregnancy remained constant between 2008 (22.9%; 95% CI = 22.14, 24.66), and 2013 (22.5%; 95% CI = 20.58, 24.50), but a significant decline was reported in 2018 (18.7%; 95% CI = 17.12, 20.46). Trends show a decrease in adolescent pregnancy across all six geopolitical zones, except for the South-East zone which reported a slight increase (0.6%). Multivariable analysis revealed that the main socioeconomic predictors across all six geopolitical zones were: poor households, increasing age, and low education. Exposure to media (watching television and reading newspapers) was reported as predictor in all regions except the North-East geopolitical zone, while all northern zones reported high levels of adolescent pregnancy in male-led households. To address adolescent pregnancy in Nigeria, there is need to promote girls’ education especially among poor households, and for the dissemination of reproductive health messages to adolescents through various forms of mass media campaign, as well as the adoption of social marketing interventions to improve sexual and reproductive health literacy.

## 1. Introduction

Adolescent pregnancy is a major health and social concern, which is associated with maternal and child morbidity and mortality [1]. To improve maternal health and reduce infant mortality, it is important to prevent pregnancy and childbirth among adolescents. According to the World Health Organization (WHO), adolescent pregnancy is the occurrence of pregnancy among young women aged 15–19 [1]. There is substantial consensus in the literature that women who become pregnant and give birth at a young age face increased risk of pregnancy-related complications and death [2,3,4,5]. The adverse social and economic consequences of adolescent pregnancy are enormous and lifelong. These consequences could lead to intergenerational cycles of poverty, suboptimal health and development, poor education, and unemployment, resulting in low socio-economic status in adulthood [4,5].

Globally, adolescent birth rates declined significantly from 65 births per 1000 female adolescents aged 15–19 in 1990 to 42 in 2018 [6,7]. However, this decline was not evenly distributed across countries, as low- and middle-income countries (LMICs) including Nigeria reported the highest birth rates while high income countries (HICs) had the lowest [8,9,10]. Given the increasing number of adolescents globally, it is anticipated that the number of adolescent pregnancies will also increase accordingly by 2030, with sub-Saharan Africa (SSA) reporting the highest increase mainly due to the high prevalence of child marriage within the region [11].

In Nigeria, the adolescent birth rate has declined from 122 births per 1000 adolescent females aged 15–19 in 2013 to 106 in 2018 [12,13]. However, this estimate is still among the highest globally, although there are intra-country variations with some regions reporting lower estimates compared with others [13]. The 2018 Nigeria Demographic Health Survey (NDHS) revealed that an estimated 1 in 5 adolescent females had begun childbearing, with rates varying from 28.5 births per 1000 adolescent females aged 15-19 years in the North-West geopolitical zone to 5.5 in the South-West [13]. To ensure no region is left behind in the attempt to reduce adolescent pregnancy in Nigeria, it is imperative that regional trends in adolescent pregnancy within the country are analysed so interventions can be targeted. One of the most important obligations of a country is to ensure safe and stable economic, social, and political progress that addresses the health and developmental needs of its younger citizens [14]. Thus, there is need to respond effectively to the health and developmental challenges faced by teenagers across all geopolitical zones in Nigeria.

Adolescent childbearing in many LMICs is often a result of early marriage [4,15], which is associated with poorer outcomes for women, including higher rates of domestic violence, reduced educational attainment, and increased levels of pregnancy-related complications, including death [15]. Early marriage, alongside other identified determinants such as poverty, place of residence, educational status of adolescents and their parents, as well as lack of exposure to timely sexuality education, predisposes adolescent girls to pregnancy [13,16]. The occurrence of early marriage is prevalent in Nigeria; however, it varis across zones with the northern geopolitical zones reporting a higher proportion than the southern zones [13,17]. Given this unequal regional distribution of early marriage, there is a possibility that adolescent pregnancy may also be concentrated in the northern region. Therefore, to reduce rates of adolescent pregnancy in Nigeria, there is need to assess the trends and ascertain the unique predisposing factors within those highly burdened zones, to inform regional level intervention.

Up-to-date understanding of the factors that lead to regional differences in adolescent fertility is essential to inform regional-level intervention policies and planning, and to improve sexual and reproductive health as well as the social and economic wellbeing of women within the country. Previous studies have examined the impacts of fertility desire, contraceptive use, marriage, and cohabitation on regional adolescent pregnancy rates in Nigeria and other SSA countries [18,19]. However, the contributing factors to regional differences in adolescent pregnancy in Nigeria are unknown. Our study aimed to address this gap by utilizing pooled data from the 2008, 2013 and 2018 NDHS to examine the regional trends and socioeconomic predictors of adolescent pregnancy in Nigeria. Findings from this study could assist in developing a deeper understanding of adolescent pregnancy in Nigeria, as well as suggest avenues where positive strategies could be deployed to reduce the occurrence of adolescent pregnancy.

## 2. Materials and Methods

### 2.1. Data Source and Sample Composition

This study is based on secondary data analysis of the 2008, 2013 and 2018 NDHSs. A total of 22,761 women aged 15–19 years were selected across the three NDHSs with 6493 women from the 2008 NDHS, 7820 from the 2013 NDHS, and 8448 from the 2018 NDHS. The NDHS is a nationally representative survey with an average response rate of 97%, which uses a multi-stage cluster sampling technique stratified by urban and rural dwellings. Further details of the survey methodology including the sampling technique have been described elsewhere [12,13,20]. The data files used in this study were individual recodes for women aged 15–19 years and are available in the public domain with all identifiable information removed [21].

### 2.2. Dependent Variable

The dependent variable of this study was adolescent pregnancy, which is the occurrence of pregnancy among young women aged 15–19. In NDHS (2008–2018), under the reproductive health section of women’s questionnaires, women were asked to report their previous pregnancy outcomes, as well as current pregnancy status. The dependent variable was measured dichotomously and coded as ‘1’ if women reported previous childbearing or were pregnant at the time of interview; and coded as ‘0’ if women had no previous childbearing or were not pregnant at the time of interview.

### 2.3. Independent Variables

**Scheme 1:**
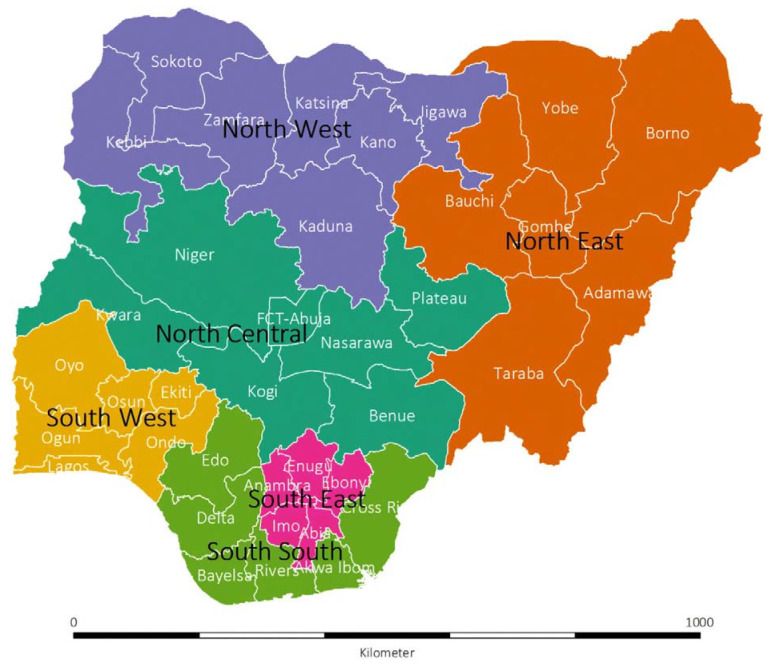
Map of Nigeria showing boundaries of six geopolitical zones, 36 states and Federal Capital Territory (FCT-Abuja).

The independent variables used in this study were based on the existing literature and the availability of data within the three NDHS (2008–2018). Since this study utilized pooled NDHS data, the year of survey (NDHS 2008, NDHS 2013, and NDHS 2018) was used as a time-dependent variable to ensure the results reported were consistent over the study period. The independent variables were broadly categorised into three groups, namely community level, household level, and individual level. The community level variables included: place of residence (1 = Urban and 2 = Rural), and state of residence across the six Nigerian geopolitical zones as shown in Scheme 1. The South-West region comprises six states (1 = Oyo, 2 = Osun, 3 = Ekiti, 4 = Ondo, 5 = Lagos, and 6 = Ogun); the North-East region consists of six states (1 = Yobe, 2 = Borno, 3 = Adamawa, 4 = Gombe, 5 = Bauchi, and 6 = Taraba); the North-West region is made up of seven states (1 = Sokoto, 2 = Zamfara, 3 = Katsina, 4 = Jigawa, 5 = Kano, 6 = Kaduna, and 7 = Kebbi); the South-East region has five states (1 = Anambra, 2 = Enugu, 3 = Ebonyi, 4 = Abia, and 5 = Imo); the South South region comprises six states (1 = Edo, 2 = Cross River, 3 = Akwa Ibom, 4 = Rivers, 5 = Bayelsa, and 6 = Delta); and the North Central region consists of seven states (1 = Niger, 2 = Abuja, 3 = Nasarawa, 4 = Plateau, 5 = Benue, 6 = Kogi, and 7 = Kwara). The household level variable included in this study was wealth index, categorised as richest, richer, middle, poorer, and poorest. Household wealth index serves as an indicator of wealth consistent with expenditure and income measures. It was represented as a score of household assets determined using the principle components analysis method (PCA) [22]. The individual level variables were age of respondent (1 = 15 years, 2 = 16 years, 3 = 17 years, 4 = 18 years, and 5 = 19 years), education level (1 = secondary or higher, 2 = primary, and 3 = no education), exposure to listening to radio (1 = yes, and 2 = no), exposure to watching television (1 = yes, and 2 = no), exposure to reading newspapers (1 = yes, and 2 = no), and sex of the household head (1 = female, and 2 = male).

### 2.4. Statistical Analysis

All statistical analyses were performed using Stata statistical software (version 14.1; StataCorp LP, College Station, Texas, USA). Survey weights in each of the NDHSs were applied due to the non-proportional allocation of the sample across regional states and variation in response rates, to ensure that the results reported were representative both at national and domain level. We first performed frequency tabulations to describe the characteristics of samples. We then estimated national and regional trends in adolescent pregnancy with 95% confidence interval (CI). Univariate logistic regression analysis was applied to measure crude odds ratio (Table S1). Multivariate logistic regression with manual backward elimination process was applied to examine the socioeconomic predictors of adolescent pregnancy in each of the six geopolitical zones of Nigeria.

### 2.5. Ethics

This study is a secondary analysis of publicly available datasets. The first author obtained permission from MEASURE DHS to download and use the 2008, 2013 and 2018 NDHS datasets, hence no ethics approval was required. NDHS is approved by the Ethics Committee of the ICF International, USA, and the National Health Research Ethics Committee of Nigeria (NHREC).

## 3. Results

### 3.1. Characteristics of Study Population

Table 1 shows the characteristics of the study population. The urban residence rates in South-West and South-East of Nigeria were substantially higher at 71.9% and 62.2%, respectively, compared to North-East (29.9%), North-West (30.7%), South South (35.8%), and North Central (28.4%). Nearly 90% of the sampled respondents from the South-West, South-East, and South South had secondary or higher education. The majority of the samples across all six zones was from male headed households (72.0% in South-West, 91.7% in North-East, 92.1% in North-West, 67.0% in South-East, 67.3% in South South, and 80.9% in North Central).

### 3.2. Trends in Adolescent Pregnancy

**Figure 1:**
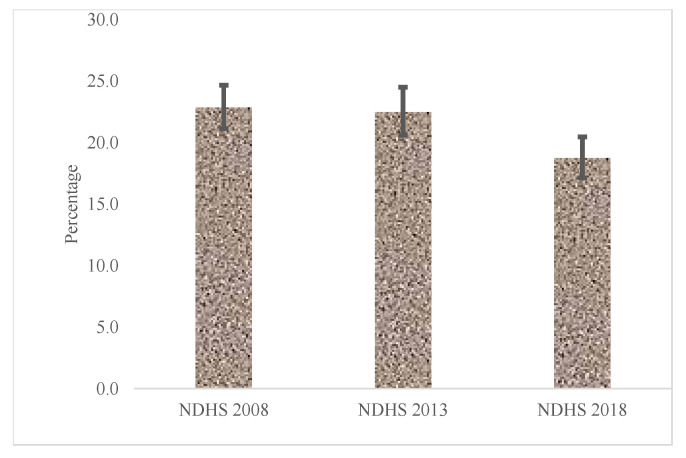
Trends in prevalence of adolescent pregnancy with 95% with CI in Nigeria, NDHS 2008–2018.

**Figure 2:**
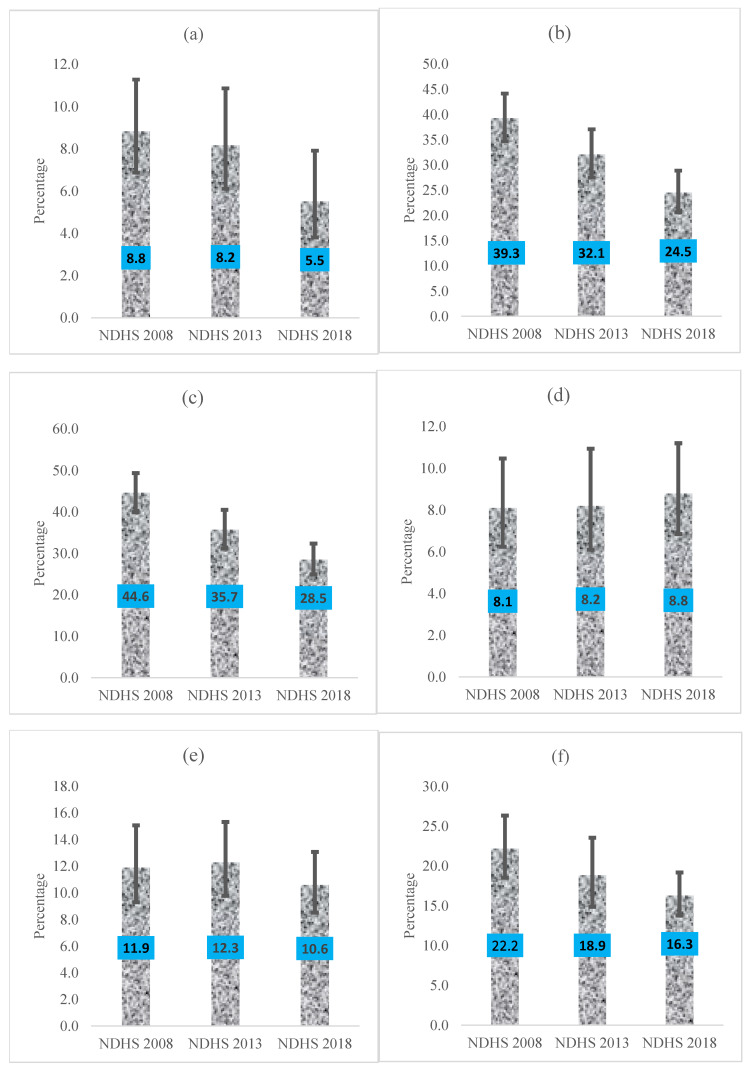
Trends in prevalence of adolescent pregnancy with 95 CI in (**a**) South West; (**b**) North East; (**c**) North West; (**d**) South East; (**e**) South South and (**f**) North central zones, Nigeria (2008–2018).

Adolescent pregnancy remained constant between 2008 (22.9%; 95% CI = 22.14, 24.66) and 2013 (22.5%; 95% CI = 20.58, 24.50), but reported a significant decline in 2018 (18.7%; 95% CI = 17.12, 20.46) as shown in Figure 1. Trends show a decrease in adolescent pregnancy across all six zones except for the South-East region, which showed a slight increase of 0.6%. A regional trend analyses showed that the zones with the highest levels of adolescent pregnancy (North-East and North-West) made substantial progress in reducing adolescent pregnancy between 2008 and 2018 (Figure 2a,b). Though some reductions in adolescent pregnancy were observed in South-West and North Central zones over the study period (2008–2018), those reductions were not statistically significant, as shown in Figure 2c,d. Furthermore, adolescent pregnancy in South-East and South South zones remained fairly constant (Figure 2e,f).

### 3.3. Regional Socioeconomic Predictors of Adolescent Pregnancy

After adjusting for potential confounding variables, the North-East, North-West, and North Central geopolitical zones showed a significant reduction in adolescent pregnancy in 2018 compared to 2008 (Table 2). In contrast, the South South region reported a significant increase in adolescent pregnancy in 2013 compared to 2008. The increase in 2018 compared to 2008 in the South South region was not statistically significant.

Across all zones, adolescent girls with lower wealth indexes (middle, poorer, and poorest) were more prone to pregnancy. Adolescent girls across all zones who reported having primary or no education were more susceptible to pregnancy compared to those who had secondary or higher levels of education. Similarly, the likelihood of adolescent pregnancy among young women in their later teenage years (16–19) was higher compared to those in their early teens (15 years).

Adolescent girls residing in rural areas in the North-East and North-West geopolitical zones had higher likelihood of being pregnant compared to their counterparts in urban areas. Adolescent girls who reported no access to television in the North-West and North Central geopolitical zones were more susceptible to pregnancy than those in other zones. Except for in the North-East and North-West, adolescent girls with no access to newspapers were more predisposed to pregnancy (in the South-West, South-East, South South, and North Central zones). Adolescent girls living in households with a male head in the North-East, North-West, and North Central geopolitical zones were more prone to adolescent pregnancy.

## 4. Discussion

Despite no progress in reducing adolescent pregnancy between 2008 and 2013, Nigeria reported a significant decline in adolescence pregnancy in 2018. However, this reduction in adolescent pregnancy was not equitable across the six geopolitical zones. Our study examined socioeconomic predictors of adolescent pregnancy in Nigeria taking into consideration the six geopolitical zones of the country. Adolescent girls from poor households, with increasing age, and those who reported a lower level of education were more susceptible to pregnancy in all six zones. Except for in the North-East, exposure to media was associated with adolescent pregnancy; and women living in a male-headed household were more prone to adolescent pregnancy in all northern zones. Furthermore, compared to northern zones, progress towards the reduction of adolescent pregnancy in the southern zones of Nigeria was not evident.

Consistent with the existing literature [5,16,23,24,25], adolescent girls from poor households residing in all geopolitical zones in Nigeria had a higher propensity to pregnancy compared to their rich counterparts. Poverty has long been established as a major underlying cause of health and social concerns [23]. Poverty has dual dynamics in adolescent pregnancy, both as a determinant and a consequence [23]. As a determinant, poverty can lead to early marriage and sexual initiation [24], and as a consequence, young girls from poor households cannot afford the cost of reproductive health services and contraceptives [24,25]. Previous studies have reported individual and environmental factors associated with adolescent pregnancy, many of which are tied to experiences of poverty or are exacerbated by poverty [16,24,25]. Poor teenagers have less autonomy, fewer opportunities, and lower expectations of future economic success, hence less reason to avoid or delay childbearing [23,25,26,27]. In addition, adolescent girls from poor households may engage in transactional sexual activity as an escape strategy from economic hardship, and this could predispose them to unintended pregnancy [28].

Alongside poverty, adolescent girls’ educational levels are a significant driver of adolescent pregnancy in Nigeria, and greatly impact their ability to negotiate sex, demand their health rights, and use preventative measures like contraception [4,24,29]. Numerous studies have reported that attaining higher levels of education deters adolescent girls from pregnancy in LMICs [28,30]. This present study contributes to the existing body of knowledge by reporting that adolescent girls with low education are more susceptible to pregnancy than their counterparts with higher educational attainment. This may be due to the fact that education increases autonomy, decision-making power, and economic independence, leading to the delay of marriage and sexual debut as well as increased knowledge of the use of contraception [29].

In this study, an increase in adolescent pregnancy with age was reported. This finding is supported by similar studies conducted in Africa [24,31,32] which also reported that as adolescent girls grew older, so did their propensity towards pregnancy. This might be because as the teenage girl advances in age, she becomes more aware of her sexuality and more exposed to sexual advances from the opposite sex. Without proper sexual education and access to contraception, the teenage girl is more prone to engaging in unprotected sex hence the increased risk of pregnancy. In addition, as teenage girls mature with age, they are more likely to be married off as young brides and housewives, given that their reproductive system is becoming more mature, and they can perform household chores without supervision. This practice increases teenage girls’ chances of becoming pregnant in their late teens, and is prevalent within some ethnic groups in Nigeria especially in the northern geopolitical zones [12,13,21]. A recent study on the spatial distribution of adolescent pregnancy in Nigeria using the 2018 NDHS showed that the northern part of Nigeria had a higher proportion of adolescent pregnancy, due to higher levels of poverty and early marriage as well as lower levels of education [33].

The current study examined the association of adolescent pregnancy with different forms of mass media exposure. Access to television was reported as protective against adolescent pregnancy in the North-West and North Central geopolitical zones but not for the southern geopolitical zones. While access to newspapers was protective in the southern zones, access to television seemed less important. These disparities across the zones in the association of mass media exposure with adolescent pregnancy invite discussion and further research around policies aimed at utilizing different forms of mass media for different zones in disseminating reproductive health messages to adolescents.

Adolescent girls need appropriate information about their emerging sexuality, including information around staying safe via the use of contraceptives and being mindful of their sexual health [34]. Media plays an important role in the lives of adolescents, providing them with opportunities for sex education. However, research has shown that in some developed countries, media content is increasingly permeated with violence and sexual references that can be highly influential during adolescent development [35,36,37]. Adolescent pregnancy thus may be correlated with the nature of sex education and the media. Hence, more frequent exposure to sexual content in the media at an early age brings increased likelihood that adolescents will engage in sexual behavior [37]. On the other hand, the media can be used to promote sex education thus preventing early pregnancy, particularly among adolescents with limited educational attainment [34]. Mass media exposure also offers improved understanding and awareness, which can foster autonomy, as well as modify attitudes, social expectations, and behaviours that could improve the well-being of adolescents [4,38]. Results from previous studies also show an inverse relationship between access to mass media and adolescent pregnancy [24,39]. In addition, exposure to mass media increases school retention which could delay marriage and sexual debut, thus preventing adolescent pregnancy [39].

In this study, adolescent pregnancy was more likely to occur in households headed by a male. This may be due to the fact that the male head is unable to engage the adolescent girl in sensitive conversations about her sexuality. Consistent with this finding is a study conducted in Nigeria that also reported lower adolescent pregnancy rates households headed by a female [40]. However, contrary to this finding is a study conducted in Malawi [41], while another study conducted in Tanzania found no association between sex of the household head and adolescent pregnancy [42]. Given these differing research findings, it could be recommended that heads of households irrespective of their sex should be educated on ways to engage adolescents in conversations around their sexuality.

### 4.1. Study Limitations and Strengths

This study has some limitations. First, due to the cross-sectional nature of the study design, this paper is limited in its ability to establish a causal relationship between the examined factors and adolescent pregnancy in Nigeria. Second, given the heterogeneity of the states and their cultures, the use of compound data to examine the socioeconomic predictors of adolescent pregnancy in Nigeria could pose a concern. However, to address this, the effect of the state-specific variable was controlled to some extent in the multilevel logistic regression analysis. Third, adolescents who reported having a previous pregnancy terminated were included as part of the measure of adolescent pregnancy, which may have led to bias in the study findings because evidence shows that data on pregnancy termination in the NDHS are often under-reported and of poor quality [43]. Finally, because this study was based on data obtained from NDHS, only adolescent girls aged 15–19 years were included. As a result, the estimation of adolescent pregnancy in this study might have been under- or overestimated.

Despite its limitations, this study also has its strengths. First, this study was based on pooled data from three consecutive population-based surveys conducted in Nigeria, in 2008, 2013 and 2018, which maximised the sample size and improved the generalizability of the results to the wider population. Second, this study applied appropriate statistical adjustments to the data obtained from nationally representative surveys, and was able to identify the most vulnerable subpopulations and zones with high levels of adolescent pregnancy as well as its trends over time across a in a large sample. Third, the findings of this study contribute to the existing body of evidence on the socioeconomic predictors of adolescent pregnancy in Nigeria.

### 4.2. Policy Implication

This present study has implications for public health and policy. Adolescent pregnancy in Nigeria varies widely across zones and within states, with the highest prevalence recorded among adolescent girls in the northern geopolitical zones of the country. Understanding the socioeconomic predictors associated with adolescent pregnancy, while controlling for regional states, adds to the existing body of evidence and could aid in effective social policy development. Successful policy development and implementation relies largely on engagement from adolescents, communities, and stakeholders in adolescent sexual and reproductive health, to drive cultural and social change. Such policies should aim at promoting girls’ education, and eradicating child marriage that puts adolescent girls at risk of pregnancy [1,44].

Our study also confirms that exposure to mass media is an overall protective factor against adolescent pregnancy. This finding highlights the opportunity for evidence-based social marketing interventions to improve sexual and reproductive health literacy. Further work is required to construct the conceptual frameworks to model the complex pathways from individual messages to changes in sexual and reproductive behaviour in a regional context.

## 5. Conclusions

Adolescent pregnancy in Nigeria varies across regions and within states. Understanding the socioeconomic predictors associated with adolescent pregnancy, while controlling for regional states, could aid in effective social policy development. This will rely largely on engagement of various stakeholders in adolescent sexual and reproductive health, to drive cultural and social change. This study shows that adolescent pregnancy results from a complex interaction of socioeconomic predictors which vary across zones. Poverty, low education, increasing adolescent age, poor exposure to mass media and sex of household head are factors that need to be considered to improve adolescent health, especially in the northern geopolitical region of Nigeria. Stakeholders should improve the socioeconomic status of the family and societal status of girls through provision of adequate girlhood education, comprehensive adolescent sexual and reproductive health education, assess to social facilities, and empowerment of the girl-child.

## Figures and Tables

**Table 1:** Characteristics of the study population in Nigeria, NDHS 2008–2018 (*N* = 22,761).

	South-West (*n* = 3656)		North-East (*n* = 3543)		North-West (*n* = 6544)		South-East (*n* = 2674)		South South (*n* = 3048)		North Central (*n* = 3296)	
Characteristics	*n*	% *	*n*	%	*n*	%	*n*	%	*n*	%	*n*	%
Year of survey												
2008	1321	36.1	856	24.2	1379	21.1	852	31.9	1127	37.0	959	29.1
2013	1121	30.7	1190	33.6	2428	37.1	894	33.4	1033	33.9	1154	35.0
2018	1215	33.2	1497	42.3	2737	41.8	928	34.7	888	29.1	1183	35.9
Types of residence												
Urban	2629	71.9	1059	29.9	2012	30.7	1662	62.2	1092	35.8	936	28.4
Rural	1027	28.1	2484	70.1	4532	69.3	1011	37.8	1956	64.2	2360	71.6
State of residence												
State 1	657	18.0	640	18.1	573	8.8	637	23.8	455	14.9	671	20.4
State 2	570	15.6	851	24.0	693	10.6	560	20.9	357	11.7	176	5.3
State 3	278	7.6	518	14.6	1081	16.5	618	23.1	558	18.3	316	9.6
State 4	480	13.1	334	9.4	670	10.2	344	12.9	344	25.4	442	13.4
State 5	1286	35.2	724	20.4	1704	26.0	515	19.3	515	9.1	785	23.8
State 6	385	10.5	476	13.4	1259	19.2				20.5	528	16.0
State 7					565	8.6					378	11.5
Wealth index												
Richest	1727	47.2	249	7.0	634	9.7	707	26.4	911	29.9	493	15.0
Richer	1037	28.4	462	13.0	980	15.0	785	29.4	949	31.1	667	20.2
Middle	522	14.3	612	17.3	1244	19.0	720	26.9	748	24.5	902	27.4
Poorer	285	7.8	903	25.5	1924	29.4	328	12.3	337	11.1	746	22.6
Poorest	86	2.3	1317	37.2	1760	26.9	134	5.0	104	3.4	488	14.8
Age of respondents												
15	918	25.1	856	24.2	1685	25.8	627	23.4	756	24.8	811	24.6
16	751	20.6	656	18.5	1135	17.4	515	19.3	605	19.9	600	18.2
17	664	18.2	656	18.5	1212	18.5	498	18.6	502	16.5	557	16.9
18	732	20.0	906	25.6	1649	25.2	583	21.8	641	21.0	791	24.0
19	591	16.2	469	13.2	862	13.2	452	16.9	544	17.9	535	16.2
Education level												
Secondary or higher	3219	88.0	1234	34.8	2383	36.4	2383	89.1	2722	89.3	2081	63.1
Primary	312	8.5	512	14.5	773	11.8	275	10.3	299	9.8	612	18.6
No education	125	3.4	1797	50.7	3388	51.8	16	0.6	27	0.9	603	18.3
Access to radio												
Yes	2923	80.0	1206	34.0	3315	50.7	2029	75.9	2148	70.5	1881	57.1
No	729	19.9	2331	65.8	3219	49.2	640	23.9	894	29.3	1411	42.8
Access to television												
Yes	3140	85.9	979	27.6	2125	32.5	2005	75.0	2507	82.2	1781	54.0
No	509	13.9	2558	72.2	4408	67.4	664	24.8	534	17.5	1507	45.7
Access to newspapers												
Yes	1146	31.3	287	8.1	816	12.5	1047	39.2	958	31.4	595	18.0
No	2503	68.5	3229	91.1	5693	87.0	1622	60.7	2082	68.3	2678	81.3
Sex of household head												
Female	1023	28.0	294	8.3	519	7.9	883	33.0	997	32.7	630	19.1
Male	2633	72.0	3249	91.7	6025	92.1	1791	67.0	2051	67.3	2666	80.9

SouthWest states (1 = Oyo, 2 = Osun, 3 = Ekiti, 4 = Ondo, 5 = Lagos, and 6 = Ogun); North-East states (1 = Yobe, 2 = Borno, 3 = Adamawa, 4 = Gombe, 5 = Bauchi, and 6 = Taraba); North-West States (1 = Sokoto, 2 = Zamfara, 3 = Katsina, 4 = Jigawa, 5 = Kano, 6 = Kaduna, and 7 = Kebbi); South-East states (1 = Anambra, 2 = Enugu, 3 = Ebonyi, 4 = Abia, and 5 = Imo); South South States (1 = Edo, 2 = Cross River, 3 = Akwa Ibom, 4 = Rivers, 5 = Bayelsa, and 6 = Delta); North Central States (1 = Niger, 2 = Abuja, 3 = Nasarawa, 4 = Plateau, 5 = Benue, 6 = Kogi, and 7 = Kwara). * Percentage does not add up to 100 because of missing values.

**Table 2:** Adjusted Odds Ratio (aOR) and 95% CI for socioeconomic predictors of adolescent pregnancy by region, NDHS 2008–2018.

	South-West		North-East		North-West		South-East		South South		North Central	
Study Variables	aOR (95% CI)	*p*-Value	aOR (95% CI)	*p*-Value	aOR (95% CI)	*p*-Value	aOR (95% CI)	*p*-Value	aOR (95% CI)	*p*-Value	aOR (95% CI)	*p*-Value
Year of survey												
2008			Reference		Reference				Reference		Reference	
2013			0.74 (0.55, 1.00)	0.053	0.90 (0.72, 1.13)	0.378			1.44 (1.01, 2.05)	0.045	0.99 (0.71, 1.39)	0.973
2018			0.58 (0.44, 0.76)	<0.001	0.62 (0.49, 0.77)	<0.001			1.12 (0.77, 1.62)	0.561	0.74 (0.56, 0.98)	0.038
Types of residence												
Urban			Reference		Reference							
Rural			2.19 (1.59, 3.02)	<0.001	2.02 (1.48, 2.77)	<0.001						
State of residence												
State 1	Reference		Reference		Reference				Reference		Reference	
State 2	0.37 (0.21, 0.66)	0.001	1.51 (0.99, 2.30)	0.058	1.31 (0.90, 1.89)	0.154			2.88 (1.71, 4.85)	<0.001	0.83 (0.50, 1.37)	0.464
State 3	0.96 (0.53, 1.75)	0.899	1.67 (1.08, 2.60)	0.022	1.88 (1.36, 2.59)	<0.001			3.15 (1.83, 5.43)	<0.001	0.96 (0.62, 1.47)	0.839
State 4	0.85 (0.49, 1.47)	0.559	1.93 (1.30, 2.86)	0.001	1.17 (0.84, 1.64)	0.348			1.83 (1.01, 3.30)	0.047	0.69 (0.42, 1.15)	0.155
State 5	0.46 (0.22, 0.93)	0.032	3.41 (2.34, 4.97)	<0.001	1.40 (1.01, 1.94)	0.041			5.15 (3.09, 8.59)	<0.001	1.57 (1.03, 2.38)	0.036
State 6	0.82 (0.46, 1.47)	0.507	1.66 (1.12, 2.44)	0.011	1.86 (1.32, 2.63)	<0.001			1.62 (0.91, 2.86)	0.098	2.49 (1.60, 3.87)	0.000
State 7					0.83 (0.59, 1.17)	0.290					1.05 (0.61, 1.79)	0.872
Wealth index												
Richest	Reference		Reference		Reference		Reference		Reference		Reference	
Richer	2.34 (1.41, 3.86)	0.001	2.64 (1.29, 5.38)	0.008	1.93 (1.19, 3.12)	0.007	2.77 (1.65, 4.63)	<0.001	2.27 (1.42, 3.62)	0.001	2.45 (1.42, 4.22)	0.001
Middle	2.25 (1.27, 4.00)	0.006	2.57 (1.18, 5.61)	0.018	2.47 (1.50, 4.06)	0.000	3.25 (1.82, 5.83)	<0.001	2.88 (1.77, 4.69)	<0.001	2.40 (1.34, 4.28)	0.003
Poorer	3.45 (1.65, 7.18)	0.001	2.98 (1.38, 6.42)	0.005	2.06 (1.23, 3.45)	0.006	3.73 (1.68, 8.28)	0.001	2.94 (1.66, 5.23)	<0.001	2.58 (1.45, 4.59)	0.001
Poorest	4.06 (1.15, 14.37)	0.030	2.22 (1.02, 4.84)	0.045	2.14 (1.27, 3.58)	0.004	2.06 (0.64, 6.58)	0.223	7.91 (3.99, 15.67)	<0.001	3.80 (1.99, 7.26)	<0.001
Age of respondents												
15	Reference		Reference		Reference		Reference		Reference		Reference	
16	2.28 (0.99, 5.28)	0.054	3.15 (2.15, 4.63)	<0.001	4.55 (3.35, 6.18)	<0.001	4.11 (1.25, 13.54)	0.020	3.45 (1.73, 6.87)	<0.001	1.96 (1.06, 3.63)	0.032
17	3.63 (1.60, 8.22)	0.002	9.84 (6.76, 14.31)	<0.001	11.29 (8.51, 14.97)	<0.001	8.8 (2.83, 27.36)	<0.001	6.91 (3.5, 13.64)	<0.001	6.65 (4.06, 10.89)	<0.001
18	11.77 (5.78, 23.93)	<0.001	16.47 (11.6, 23.38)	<0.001	21.68 (16.38, 28.69)	<0.001	24.01 (7.91, 72.93)	<0.001	10.15 (5.54, 18.61)	<0.001	15.89 (9.79, 25.79)	<0.001
19	21.68 (9.92, 47.39)	<0.001	40.12 (26.41, 60.97)	<0.001	40.81 (29.59, 56.29)	<0.001	38.22 (12.67, 115.27)	<0.001	24.05 (12.87, 44.92)	<0.001	30.75 (18.29, 51.7)	<0.001
Education level												
Secondary or higher	Reference		Reference		Reference		Reference		Reference		Reference	
Primary	4.51 (2.77, 7.32)	<0.001	3.60 (2.60, 4.97)	<0.001	4.65 (3.47, 6.24)	<0.001	2.26 (1.32, 3.85)	0.003	2.81 (1.92, 4.11)	<0.001	2.48 (1.74, 3.54)	<0.001
No education	5.83 (3.04, 11.18)	<0.001	7.9 (5.86, 10.64)	<0.001	8.16 (6.23, 10.68)	<0.001	3.84 (1.47, 10.02)	0.006	1.80 (0.54, 5.96)	0.334	5.57 (3.83, 8.10)	<0.001
Access to Radio												
Yes												
No												
Access to Television												
Yes					Reference						Reference	
No					1.61 (1.25, 2.07)	<0.001					1.48 (1.10, 1.99)	0.010
Access to Newspaper												
Yes	Reference						Reference		Reference		Reference	
No	2.13 (1.32, 3.46)	0.002					1.95 (1.29, 2.94)	0.002	2.20 (1.45, 3.34)	<0.001	2.55 (1.58, 4.10)	<0.001
Sex of household head												
Female			Reference		Reference						Reference	
Male			2.79 (1.76, 4.43)	<0.001	1.67 (1.26, 2.21)	<0.001					2.06 (1.45, 2.94)	<0.001

CI = Confidence Interval.

## Data Availability

The NDHS datasets can be found at https://dhsprogram.com/data/available-datasets.cfm (accessed on 10 November 2021).

## Supplementary Materials

The following supporting information can be downloaded at: https://www.mdpi.com/article/10.3390/ijerph19138222/s1, Table S1: Crude odds ratio with 95% CI for socioeconomic predictors of adolescent pregnancy by region, NDHS 2008–2018.
