# Supplementary material for: Regional Trends and Socioeconomic Predictors of Adolescent Pregnancy in Nigeria: A Nationwide Study
Source: Int J Environ Res Public Health. 2022 Jul 5;19(13):8222. doi: 10.3390/ijerph19138222 (PMC9266692; doi:10.3390/ijerph19138222)
Supplement: Supplementary file 1 [file ijerph-19-08222-s001.zip › ijerph-1773256-supplementary.pdf]

**Table S1.** Crude Odds Ratio (COR) with 95% CI for socioeconomic predictors of adolescent pregnancy by region, NDHS 2008-2018.

| | South West | | North East | | North West | | South East | | South South | | North Central | |
|---|---|---|---|---|---|---|---|---|---|---|---|---|
| Study variables | COR (95% CI) | p-Value | COR (95% CI) | p-Value | COR (95% CI) | p-Value | COR (95% CI) | p-Value | COR (95% CI) | p-Value | COR (95% CI) | p-Value |
| Year of survey | | | | | | | | | | | | |
| 2008 | Reference | | Reference | | Reference | | Reference | | Reference | | Reference | |
| 2013 | 0.92 (0.60, 1.40) | 0.690 | 0.73 (0.55, 0.97) | 0.030 | 0.69 (0.52, 0.91) | 0.010 | 1.01 (0.66, 1.55) | 0.950 | 1.04 (0.71, 1.51) | 0.840 | 0.81 (0.57, 1.17) | 0.270 |
| 2018 | 0.60 (0.37, 0.97) | 0.040 | 0.50 (0.37, 0.68) | <0.001 | 0.50 (0.38, 0.64) | <0.001 | 1.09 (0.74, 1.62) | 0.660 | 0.88 (0.61, 1.26) | 0.480 | 0.68 (0.51, 0.92) | 0.010 |
| Types of residence | | | | | | | | | | | | |
| Urban | Reference | | Reference | | Reference | | Reference | | Reference | | Reference | |
| Rural | 2.28 (1.60, 3.24) | <0.001 | 3.16 (2.31, 4.32) | <0.001 | 4.61 (3.49, 6.08) | <0.001 | 1.3 (0.94, 1.8) | 0.120 | 2.13 (1.49, 3.04) | <0.001 | 2.2 (1.65, 2.95) | <0.001 |
| State of residence | | | | | | | | | | | | |
| State 1 | Reference | | Reference | | Reference | | Reference | | Reference | | Reference | |
| State 2 | 0.31 (0.18, 0.54) | <0.001 | 1.00 (0.62, 1.60) | 0.990 | 0.99 (0.68, 1.45) | 0.960 | 0.99 (0.57, 1.74) | 0.980 | 4.11 (2.48, 6.84) | <0.001 | 0.23 (0.14, 0.37) | <0.001 |
| State 3 | 0.66 (0.37, 1.20) | 0.180 | 0.96 (0.62, 1.48) | 0.850 | 1.17 (0.83, 1.67) | 0.370 | 1.19 (0.74, 1.92) | 0.480 | 3.64 (2.22, 5.99) | <0.001 | 0.40 (0.26, 0.61) | <0.001 |
| State 4 | 0.73 (0.43, 1.22) | 0.230 | 1.40 (0.91, 2.14) | 0.130 | 1.08 (0.77, 1.50) | 0.650 | 1.53 (0.92, 2.55) | 0.100 | 2.27 (1.25, 4.13) | 0.010 | 0.31 (0.20, 0.49) | <0.001 |
| State 5 | 0.22 (0.12, 0.41) | <0.001 | 2.35 (1.53, 3.59) | <0.001 | 0.66 (0.46, 0.95) | 0.030 | 1.06 (0.57, 1.98) | 0.850 | 4.84 (2.97, 7.89) | <0.001 | 0.62 (0.41, 0.94) | 0.030 |
| State 6 | 0.71 (0.44, 1.17) | 0.180 | 0.90 (0.58, 1.39) | 0.630 | 0.78 (0.53, 1.15) | 0.210 | | | 1.93 (1.09, 3.42) | 0.030 | 0.50 (0.33, 0.76) | <0.001 |
| State 7 | | | | | 0.80 (0.55, 1.14) | 0.220 | | | | | 0.38 (0.22, 0.67) | <0.001 |
| Wealth index | | | | | | | | | | | | |
| Richest | Reference | | Reference | | Reference | | Reference | | Reference | | Reference | |
| Richer | 2.68 (1.67, 4.31) | <0.001 | 4.23 (2.15, 8.33) | <0.001 | 3.29 (2.06, 5.27) | <0.001 | 2.84 (1.63, 4.94) | <0.001 | 2.69 (1.73, 4.16) | <0.001 | 3.45 (2.08, 5.71) | <0.001 |
| Middle | 2.67 (1.63, 4.38) | <0.001 | 6.13 (2.96, 12.69) | <0.001 | 7.8 (4.94, 12.33) | <0.001 | 3.5 (2.02, 6.05) | <0.001 | 4.07 (2.59, 6.40) | <0.001 | 4.40 (2.63, 7.37) | <0.001 |
| Poorer | 4.96 (2.94, 8.36) | <0.001 | 10.35 (4.95, 21.62) | <0.001 | 11.79 (7.56, 18.38) | <0.001 | 3.74 (2.12, 6.59) | <0.001 | 4.29 (2.63, 6.98) | <0.001 | 6.77 (4.09, 11.2) | <0.001 |
| Poorest | 9.4 (4.47, 19.80) | <0.001 | 11.68 (5.66, 24.10) | <0.001 | 12.86 (8.34, 19.84) | <0.001 | 2.63 (1.14, 6.1) | 0.020 | 10.58 (5.47, 20.44) | <0.001 | 11.68 (6.72, 20.29) | <0.001 |
| Age of respondents | | | | | | | | | | | | |
| 15 | Reference | | Reference | | Reference | | Reference | | Reference | | Reference | |
| 16 | 2.44 (1.10, 5.39) | 0.030 | 2.71 (1.89, 3.89) | <0.001 | 3.69 (2.78, 4.88) | <0.001 | 3.98 (1.21, 13.15) | 0.020 | 2.77 (1.44, 5.31) | <0.001 | 1.72 (0.97, 3.04) | 0.060 |
| 17 | 2.66 (1.23, 5.76) | 0.010 | 7.38 (5.28, 10.31) | <0.001 | 8.47 (6.50, 11.04) | <0.001 | 7.7 (2.55, 23.22) | <0.001 | 4.94 (2.66, 9.18) | <0.001 | 5.01 (3.15, 7.98) | <0.001 |
| 18 | 9.58 (4.92, 18.65) | <0.001 | 10.68 (7.85, 14.52) | <0.001 | 15.13 (11.69, 19.59) | <0.001 | 21.22 (7.25, 62.11) | <0.001 | 7.52 (4.30, 13.14) | <0.001 | 10.85 (6.88, 17.12) | <0.001 |
| 19 | 13.78 (7.05, 26.93) | <0.001 | 16.97 (11.83, 24.36) | <0.001 | 18.85 (14.16, 25.09) | <0.001 | 30.22 (10.3, 88.69) | <0.001 | 15.67 (8.89, 27.62) | <0.001 | 17.14 (10.7, 27.46) | <0.001 |
| Education level | | | | | | | | | | | | |
| Secondary or higher | Reference | | Reference | | Reference | | Reference | | Reference | | Reference | |
| Primary | 4.40 (2.84, 6.82) | <0.001 | 3.91 (2.88, 5.31) | <0.001 | 5.30 (4.09, 6.88) | <0.001 | 1.88 (1.24, 2.84) | <0.001 | 3.08 (2.27, 4.18) | <0.001 | 2.95 (2.21, 3.93) | <0.001 |
| No education | 9.78 (6.48, 14.76) | <0.001 | 7.29 (5.59, 9.50) | <0.001 | 9.66 (7.84, 11.91) | <0.001 | 5.83 (2.1, 16.24) | <0.001 | 3.54 (1.32, 9.47) | 0.010 | 7.39 (5.4, 10.12) | <0.001 |
| Access to Radio | | | | | | | | | | | | |

| | | | | | | | | | | | | |
|---|---|---|---|---|---|---|---|---|---|---|---|---|
| Yes | Reference | | Reference | | Reference | | Reference | | Reference | | Reference | |
| No | 1.09 (0.74, 1.61) | 0.660 | 1.26 (1.05, 1.51) | 0.010 | 1.02 (0.86, 1.20) | 0.850 | 1.23 (0.88, 1.71) | 0.220 | 1.63 (1.28, 2.08) | <0.001 | 1.97 (1.62, 2.41) | <0.001 |
| Access to Television | | | | | | | | | | | | |
| Yes | Reference | | Reference | | Reference | | Reference | | Reference | | Reference | |
| No | 2.49 (1.72, 3.60) | <0.001 | 2.81 (2.16, 3.66) | <0.001 | 2.94 (2.41, 3.59) | <0.001 | 1.57 (1.15, 2.15) | 0.010 | 2.23 (1.68, 2.97) | <0.001 | 3.01 (2.38, 3.81) | <0.001 |
| Access to Newspaper | | | | | | | | | | | | |
| Yes | Reference | | Reference | | Reference | | Reference | | Reference | | Reference | |
| No | 3.06 (1.96, 4.77) | <0.001 | 4.61 (2.83, 7.51) | <0.001 | 5.49 (3.94, 7.66) | <0.001 | 1.94 (1.37, 2.75) | <0.001 | 2.70 (1.90, 3.82) | <0.001 | 4.97 (3.25, 7.60) | <0.001 |
| Sex of household head | | | | | | | | | | | | |
| Female | Reference | | Reference | | Reference | | Reference | | Reference | | Reference | |
| Male | 1.44 (1.00, 2.07) | 0.050 | 4.00 (2.63, 6.09) | <0.001 | 1.88 (1.37, 2.58) | <0.001 | 0.82 (0.59, 1.14) | 0.250 | 0.86 (0.65, 1.14) | 0.300 | 2.63 (1.96, 3.54) | <0.001 |

COR=Crude Odds Ratio, CI=Confidence Interval
